# A Study of Memory Anomalies in OpenMP Applications

**DOI:** 10.1007/978-3-030-58144-2_21

**Published:** 2020-08-03

**Authors:** Lechen Yu, Joachim Protze, Oscar Hernandez, Vivek Sarkar

**Affiliations:** 8 Texas Advanced Computing Center (TACC), Austin, TX USA; 9 Lawrence Livermore National Laboratory, Livermore, CA USA; 10 Texas Advanced Computing Center (TACC), Austin, TX USA; 11 RWTH Aachen University, Aachen, Germany; 12 Georgia Institute of Technology, Atlanta, GA USA; 13 RWTH Aachen University, Aachen, Germany; 14 Oak Ridge National Laboratory, Oak Ridge, TN USA

**Keywords:** Memory anomalies, OpenMP, Tools

## Abstract

Incorrect usage of OpenMP constructs may cause different kinds of defects in OpenMP applications. Most of the existing work focuses on concurrency bugs such as data races and deadlocks, since concurrency bugs are difficult to detect and debug. In this paper, we discuss an under-examined defect in OpenMP applications: memory anomalies. These occur when the application issues illegal memory accesses that may result in a non-deterministic result or even a program crash. Based on the latest OpenMP 5.0 specification, we analyze some OpenMP usage errors that may lead to memory anomalies. Then we illustrate three kinds of memory anomalies: use of uninitialized memory (UUM), use of stale data (USD), and use after free (UAF). While all three anomalies can occur in sequential programs, their manifestations in parallel OpenMP programs can be different, and debugging such anomalies in the context of parallel programs also imposes an additional complexity relative to sequential programs. To measure the effectiveness of memory anomaly detectors on OpenMP applications, we have evaluated three state-of-the-art tools with a group of micro-benchmarks. These micro-benchmarks are either selected from the DRACC benchmark suite or constructed from our own experience. The evaluation result shows that none of these tools can currently handle all three kinds of memory anomalies.

## Introduction

  OpenMP is the de facto standard for on-node parallel programming in HPC. It provides a unified execution model for parallel applications written in C/C++ and Fortran. OpenMP integrates multiple parallel paradigms in its execution model, including task parallelism, single program multiple data (SPMD), and heterogeneous parallelism. Each parallel paradigm is implemented by a set of high-level constructs, and programmers can mix these paradigms in a single OpenMP application. The unified execution model can help programmers fully utilize computing resources in a single mainstream programming system.

**Fig. 1.**
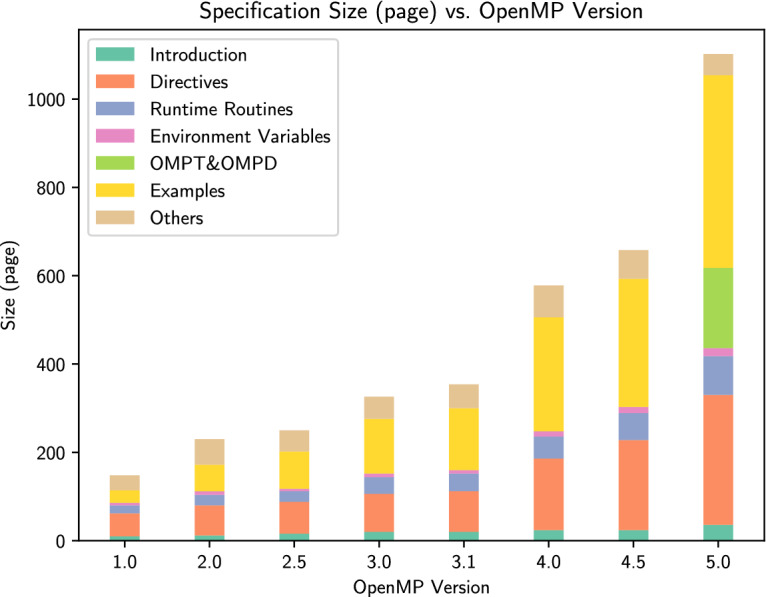
**A comparison among the size of OpenMP specifications:** OpenMP 1.0 and 2.0 have separate documents for Fortran and C/C++, so we show the accumulated number of pages for each section. Starting from OpenMP 4.0, the *examples* appendix became a standalone document; for comparison we include the *OpenMP examples* document into the page count.

The unified execution model brings about high expressibility, but it also increases the complexity of OpenMP. According to Fig. 1, every time a new paradigm is introduced into OpenMP, the size of the specification increases significantly, e.g., when task parallelism was added in OpenMP 3.0, and heterogeneous parallelism was added in OpenMP 4.0 and 4.5). The latest OpenMP 5.0 [20] specification is now already larger than 600 pages. Correctly understanding the semantics of OpenMP constructs becomes challenging even for experienced programmers, which makes OpenMP applications error-prone. There has been a large amount of prior work related to the correctness of OpenMP applications, including tools [3–5, 7, 11, 16, 21, 24, 26], benchmarks [1, 9, 12], and empirical studies [13, 14, 17]. Most of this past work focuses on concurrency bugs such as data races, deadlocks, and atomicity violations, since debugging concurrency bugs are notoriously difficult for programmers. However, other types of bugs may also be manifest at runtime, for example, memory anomalies [2]. To the best of our knowledge, there has been no comprehensive study thus far to analyze memory anomalies in OpenMP applications.

Since C/C++ delegates the responsibility of memory management to programmers, memory anomaly is a common problem in C/C++ applications. It happens when the program issues memory operations to certain memory regions that are not ready for memory access (e.g., freed or uninitialized memory locations). Memory anomalies can be further classified into sub-categories according to their runtime behavior, such as the use of uninitialized memory (UUM), use of stale data (USD), and use after free (UAF). For C/C++ applications, memory anomalies have been well-studied, and several dynamic detectors are available. Prior work refers to memory anomalies also as memory vulnerabilities and memory errors, and all these terms are used alternatively for the same group of defects.

In this paper, we investigate the impact of memory anomalies on OpenMP programs. We have conducted a study of memory anomalies in OpenMP applications, guided by the following research questions:

- **RQ1 (Bug patterns):** What are the common bug patterns of memory anomalies in OpenMP? What are the root causes of these memory anomalies?
- **RQ2 (Bugfix):** How to fix memory anomalies in an OpenMP application?
- **RQ3 (Tool effectiveness):** What is the effectiveness of state-of-art memory anomaly detectors on OpenMP applications?

To answer RQ1–RQ3, we have examined the semantics of OpenMP constructs that are related to memory allocation/deallocation. We found that memory anomalies may result from incorrect settings of the data-sharing attribute and map-type. These two properties indicate a variable’s visibility among threads and devices. If these two properties are not explicitly set up by programmers, OpenMP will apply a complex rule-based mechanism to determine them at runtime. To illustrate probable memory anomalies in OpenMP applications and analyze their relationship with data-sharing attribute and map-type, we constructed a group of benchmarks with memory anomalies, based on our own experience as well as collected memory anomalies from open-source benchmarks, We leveraged these micro-benchmarks to evaluated three state-of-art memory anomaly detectors: AddressSanitizer (ASan) [22], MemorySanitizer (MSan) [23], and Valgrind [18]. The evaluation result shows that the three tools can precisely detect a subset of memory anomalies in OpenMP applications, and none of them can tackle all three kinds of memory anomalies.

The rest of this paper is organized as follows. Section 2 introduces data-sharing attributes and map-types, as well as the rule-based inference mechanism. Section 3 illustrates UUM, USD, and UAF with some buggy examples. In Sect. 4, we present an evaluation of three memory anomaly detectors and discuss the evaluation results. We summarize related work in Sect. 5, and finally, in Sect. 6, we conclude and mention possible directions for future work.

## OpenMP’s Data-Sharing Attribute and Map-Type

In this section, we outline the data-sharing attribute and map-type. These two properties are commonly used in OpenMP to specify variables’ visibility. Due to their effect on memory access, incorrect settings of data-sharing attribute or map-type may result in unexpected behavior, which is the major source of memory anomalies in OpenMP applications.

### Semantics

According to the OpenMP specification [20], an OpenMP application starts execution on the host (CPU) and may offload computations and data to other target devices (accelerators). Each device (host and target devices) has a group of threads and an independent memory space, and threads are not migratable among devices. By invoking corresponding OpenMP constructs, an OpenMP application can utilize any supported parallel paradigm (tasking constructs for task parallelism, parallel and worksharing constructs for SPMD, target constructs for heterogeneous parallelism). Multiple paradigms may be cooperatively applied in a single OpenMP application, to separate the workload into available computing resources properly.

OpenMP uses the data-sharing attribute to define the intra-device visibility of allocated variables. The data-sharing attribute allows two types of visibility: *private* and *shared*. Memory accesses to a shared variable operate on the variable’s original memory region, and proper synchronization needs to be carried out to avoid race conditions. For a private variable, each (implicit or explicit) task creates a local instance of the variable. Memory accesses in a particular thread are performed on the local instance, which is not visible to other threads. In addition, there are two variants of the private visibility: *first-private* and *last-private*. The former declares the initialization of the local instance by the original variable. The latter denotes that the original variable is to be updated by the last assigned value.

When it comes to heterogeneous parallelism, a variable may have multiple storages on different devices. Since a device’s memory space may not be visible to threads on other devices, programmers need to specify data transfers through map-types to synchronize these independent storages. For variables accessed by target regions (code sections executing on the target device), their map-types needs to be correctly set up to avoid potential memory anomalies. In total, there are five available options:

- *to*: copy the variable from host to target device
- *alloc*: allocate an uninitialized storage on the target
- *from*: copy the variable from the target device to host
- *delete/release*: deallocate the storage on the target device
- *tofrom*: a combination of ‘to’ and ‘from’.

During the execution of an OpenMP application, the underlying OpenMP runtime automatically issues necessary data transfers according to the specified map-types. For map-type ‘to’ and ‘alloc’, the corresponding data transfer is carried out before the target region, while for map-type ‘from’, ‘delete’, and ‘release’, the data transfer is inserted at the end of the target region.

### Determining Data-Sharing Attribute and Map-Type

According to the OpenMP specification, specifying the data-sharing attribute for every variable is not mandatory. OpenMP defines a set of inference rules to determine the data-sharing attribute at runtime, which helps reduce the programming effort. Table 1 lists all inference rules for the data-sharing attribute. The first column describes these rules’ preconditions. For a particular variable, the data-sharing attribute is determined by the variable type, enclosing construct, and the default value. The second and third columns show each rule’s type and output, where PRE, EXP, and IMP stand for ‘predetermined’, ‘explicit’, and ‘implicit’, respectively.

For certain OpenMP constructs, the specification defines a number of predetermined rules for associated data sharing attributes. Most of these rules cannot be overwritten by programmer-specified data-sharing attributes^1^. If no predetermined rules match and the data-sharing attribute is unspecified in the program, OpenMP turns to implicit rules. The output of implicit rules depends on the default data-sharing attribute. When no default clauses present in an OpenMP application, OpenMP sets the default data-sharing attribute to ‘shared’ for a parallel construct, and ‘first-private’ for a tasking and target constructs.

The reduction clause is a special case in terms of data-sharing attributes. For each variable appearing in a reduction clause, the OpenMP implementation needs to allocate a local instance for each task. At the end of the scope, the runtime combines the original value with each local instance’s final value. Therefore, the data-sharing semantics of the reduction clause is a combination of first-private and last-private.

OpenMP also defines inference rules for map-type. Compared to the data-sharing attribute, map-type’s inference rules are much more straightforward. For a variable accessed by a target region and having no explicit map-type: a) If the variable is a scalar, then it is not mapped, and its data-sharing attribute is set to ‘first-private’ by the predetermined inference rule; b) Otherwise, it is mapped to the target device with map-type ‘tofrom’.

**Table 1.** Inference rules for data-sharing attributes

Precondition		Rule type	Data-sharing attribute
Declared inside a construct		PRE	private
Static class member, objects with dynamic storage duration		PRE	shared
A loop iteration variable in a for, parallel for, task loop, or distribute construct		PRE	private
A loop iteration variable in a simd or loop construct		PRE	last-private
Listed in a reduction clause		EXP	private
Listed in a data-sharing attribute clause (programmer-specified data-sharing attribute)		EXP	Determined by the clause
An unmapped variable in a target construct		IMP	first-private
default clause is not present	In a parallel construct	IMP	shared
	In a task, taskloop, target, target enter data, target exit data, target update construct	IMP	first-private
default clause is present	In a parallel and teams construct	IMP	Determined by default clause
	In a task, taskloop, target, target enter data, target exit data, target update construct	IMP	

## Memory Anomalies in OpenMP Applications

To answer the three research questions proposed in Sect. 1, we have conducted a study on OpenMP to find out probable memory anomalies. By scrutinizing the semantics of OpenMP constructs, we found that incorrect OpenMP usage may lead to three kinds of memory anomalies: use of uninitialized memory (UUM), use after free (UAF), and use of stale data (USD). We devised a few buggy examples from our own experiences as well as collected defects in open-source benchmarks. In this section, we illustrate these examples and talk about our findings related to RQ1 and RQ2.

### Use of Uninitialized Memory

Programmers may specify incorrect data-sharing attributes in an OpenMP application. Typically these incorrect settings will introduce bugs. If a private variable is set to be shared, then a data race may occur. If a shared variable is set to private, then subsequent memory accesses to this variable may become UUMs.

**Fig. 2.**
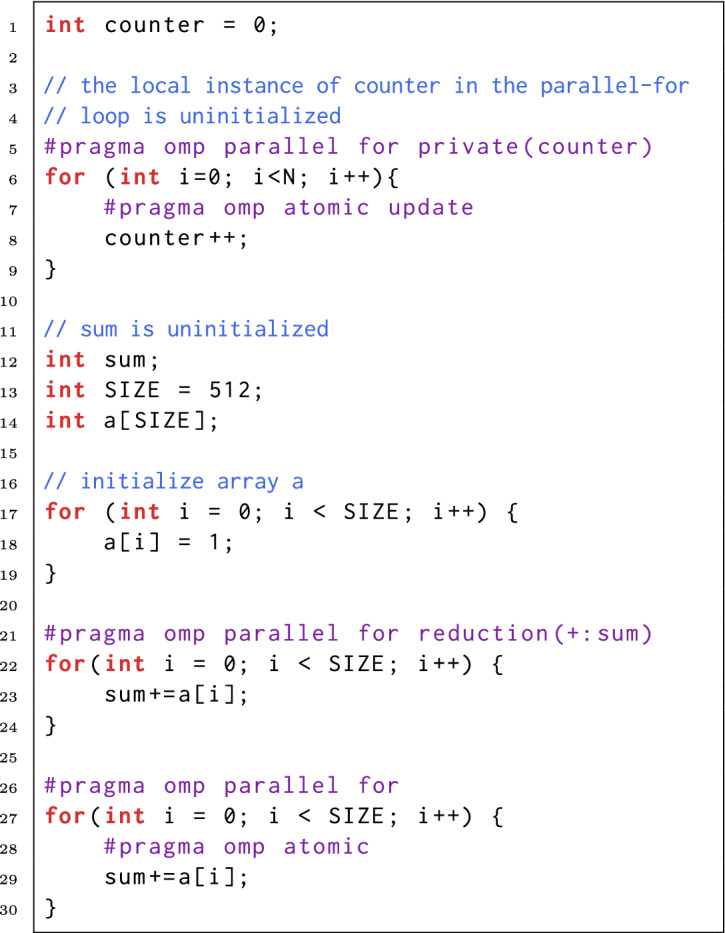
UUMs in parallel for constructs

In Fig. 2, we present a few examples of UUMs. The first UUM is in line 8, and its root cause is the incorrect data-sharing attribute set in line 5. This error can be fixed by setting the data-sharing attribute to ‘shared’, removing the private clause, or using a sum reduction.

**Fig. 3.**
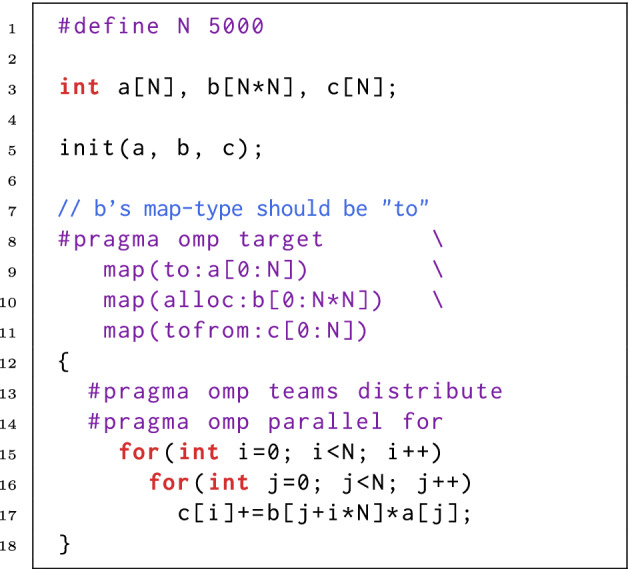
A UUM resulting from incorrect map-type

Incorrect map-type is another source of UUMs. We show an example in Fig. 3. In line 10, the map-type is set to ‘alloc’, so that a storage for array b is allocated but not initialized on the target device. The subsequent read in line 17 is a UUM since b is uninitialized. To fix this error, b’s map-type should be set to ‘to’ or ‘tofrom’.

In some cases, OpenMP constructs are not the root cause of memory anomalies, but they make memory anomaly detection difficult, such as the two UUMs in Fig. 2 (in line 23 and 29). These two UUMs are associated with OpenMP constructs (reduction clause and atomic construct). The memory anomaly detector must take OpenMP constructs into account, and correctly model their semantics. Otherwise, the memory anomaly detector may cause false alarms or miss some bugs when examining an OpenMP application.

### Use After Free

**Fig. 4.**
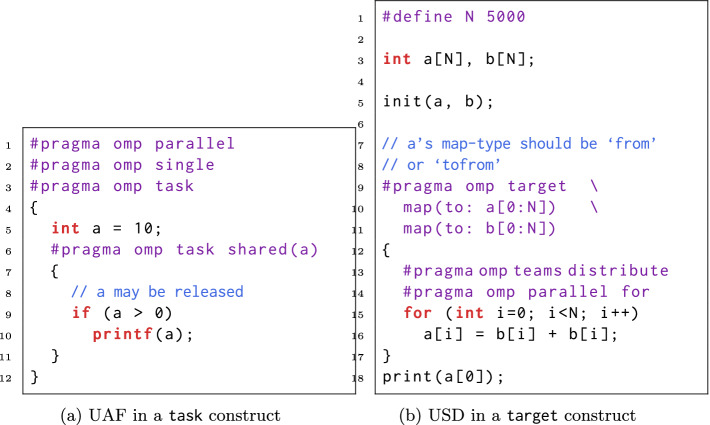
UAF and USD

Figure 4a displays an example of UAF. The outer task spawns the inner task in line 6, and the inner task tries to access a in line 9 and 10. However, the generating task does not suspend after creating the inner task. It may terminate before the inner task issues the two read operations. So that these two reads become UAF since a has been deallocated. To avoid such errors, programmers need to understand a variable’s lifetime correctly, and pay special attention to shared variables.

### Use of Stale Data

USDs are defined against the dataflow. For example, Cilk proposed a notion *serial elision* that a parallel application’s dataflow needs to be consistent with its sequential version [10]. Based on the serial elision property, a USD happens when a Cilk application generates an inconsistent dataflow with the sequential version. However, in OpenMP applications, the serial elision property may not hold since the specification does not enforce it.

We redefine USDs in OpenMP applications with regard to the uncertainty of unified memory. An OpenMP implementation may activate unified memory when executing target regions. By the requires(unified_shared_memory) directive, programmers can notify the OpenMP implementation that unified memory is indispensable for the correctness of the results. Otherwise, the OpenMP implementation assumes that the application always generates correct and consistent results regardless of unified memory.

To distinguish the executions with and without unified memory, we refer to the execution with unified memory enabled as the *unified memory version*, and the execution without unified memory as the *original version*. USDs are defined against these two versions:

#### Definition 1 (Use of Stale Data)

For an OpenMP application without require directives, A USD happens if the original version has inconsistent dataflow with the unified memory version.

Similar to UUMs, USDs may also arise from incorrect data-sharing rules and map-types. Figure 4b shows an example of USD. For the unified memory version, map clauses are optional since the effect of memory accesses are observable to any devices. So that the print statement in line 18 returns the value written by line 16. For the original version, each device’s memory space is isolated. Because array a’s map-type is set to ‘to’ in line 10, the value of array a on the target device is not copied back to the host. Thus the print statement returns the initial value of a[0], which is inconsistent with the unified memory version. To fix this USD, array a’s map-type should be set to ‘from’ or ‘tofrom’.

## Evaluation of Memory Anomaly Detectors

In this section, we try to answer RQ3. We present the evaluation of three state-of-the-art memory anomaly detectors. All experiments were performed on a compute node of CLAIX cluster running CentOS 7, and all benchmarks are compiled with LLVM 10.0.

### Memory Anomaly Detectors and Benchmarks

We evaluated three dynamic memory anomaly detectors and compared their effectiveness. All three tools are commonly used when debugging C/C++ applications. ASan is a dynamic analysis tool developed by Google. It can detect a variety of memory anomalies related to pointer dereference (e.g., UAF, buffer overflow). MSan is another dynamic detector from Google. Is uses the same infrastructure as ASan, but is designed to detect UUMs. Valgrind is a debugging and profiling tool suite running on top of a dynamic instrumentation framework. The memcheck tool is capable of detecting memory anomalies and memory leaks. In our evaluation, we used ASan and MSan implemented in LLVM 10.0, and the memcheck tool in Valgrind 3.14.0.

To measure the effectiveness of the three detectors, we constructed a benchmark suite consisting of 22 small OpenMP applications. 15 benchmarks were chosen from DRACC [1] and each of them contains a memory anomaly caused by incorrect data transfer. The remaining seven benchmarks are created by ourselves. Since in DRACC there exist no memory anomalies resulting from the data-sharing attribute, we devised the seven benchmarks based on our experience on OpenMP to improve the benchmark suite’s coverage^2^.

### Evaluation Result

Table 2 lists the evaluation result on the 15 DRACC benchmarks. Columns 1–2 record the benchmark name and the error type. Columns 3–5 indicate the result of memory anomaly detection. Here we apply the same group of notions used in [12] and [5], where TP denotes that the tool correctly reports the error, and FN denotes the tool misses the known error.

Out of the 15 known errors, ASan and MSan successfully reported 6 and 5 memory anomalies. ASan detected all buffer overflows and MSan pinpointed all UUMs. These results match our expectation because their detection algorithms are customized for a specific set of memory anomalies. Valgrind detected 9 out of 15 memory anomalies, which outperforms the other two detectors. However, Valgrind’s bug report is quite noisy. It reported a large number of memory anomalies in system libraries, making the bug report difficult to understand. On the other hand, all three detectors missed USDs. To detect USDs, memory anomaly detectors need to model an OpenMP applications’ dataflow correctly. Currently, none of the three detectors takes the dataflow into account.

**Table 2.** Evaluation result on DRACC benchmarks

Benchmark	Memory anomaly	Effectiveness		
		ASan	MSan	Valgrind
DRACC_OMP_022	UUM	FN	TP	FN
DRACC_OMP_023	Buffer overflow	TP	FN	TP
DRACC_OMP_024	UUM	FN	TP	FN
DRACC_OMP_025	Buffer overflow	TP	FN	TP
DRACC_OMP_026	USD	FN	FN	FN
DRACC_OMP_027	USD	FN	FN	FN
DRACC_OMP_028	Buffer overflow	TP	FN	TP
DRACC_OMP_029	Buffer overflow	TP	FN	TP
DRACC_OMP_030	Buffer overflow	TP	FN	TP
DRACC_OMP_031	Buffer overflow	TP	FN	TP
DRACC_OMP_032	USD	FN	FN	FN
DRACC_OMP_033	USD	FN	FN	FN
DRACC_OMP_049	UUM	FN	TP	TP
DRACC_OMP_050	UUM	FN	TP	TP
DRACC_OMP_051	UUM	FN	TP	TP
Overall		6/15	5/15	9/15

Table 3 lists the evaluation result on the remaining seven benchmarks. Similar to Table 2, Valgrind outperforms ASan and MSan on the number of detected errors. In addition, ASan failed to detect a UAF, and MSan missed two UUMs in DAS_OMP_003 and DAS_OMP_005. These three memory anomalies are expected to be detected. One possible reason is that ASan and MSan handle some OpenMP constructs improperly, since both benchmarks use the atomic construct to protect memory accesses to an uninitialized variable.

**Table 3.** Evaluation result on additional benchmarks

Benchmark	Memory anomaly	Effectiveness		
		ASan	MSan	Valgrind
DSA_OMP_001	UUM	FN	TP	TP
DSA_OMP_002	UUM	FN	TP	TP
DSA_OMP_003	UUM	FN	FN	TP
DSA_OMP_004	UUM	FN	TP	TP
DSA_OMP_005	UUM	FN	FN	TP
DSA_OMP_006	UAF	FN	TP	FN
DSA_OMP_007	USD	FN	FN	FN
Overall		0/7	4/7	5/7

### Lesson Learned

The evaluation result indicates that none of the three tools can handle all memory anomalies. Because different kinds of memory anomalies have distinct runtime behavior, it is challenging for a single detector to capture all memory anomalies. To reduce the possibility of missing memory anomalies, programmers could apply multiple detectors simultaneously when debugging an OpenMP application.

## Related Work

In this section, we relate our study on OpenMP to representative work in memory anomaly detection and bug studies.

### Memory Anomaly Detection

Memory anomalies in C/C++ applications have been comprehensively studied in the past decades, and a large number of memory anomaly detectors have been proposed [6, 8, 18, 22, 23]. These detectors monitor the execution of a C/C++ application, recording relevant metadata for each memory locations (e.g., status, base address, bound). When a memory location is accessed, the tools examine associated metadata to check the legality of the memory access. These detectors can pinpoint memory anomalies at runtime, with acceptable overhead to the program execution. When applied to OpenMP applications, these detectors may generate false positives and false negatives due to the misunderstanding of OpenMP constructs.

To the best of our knowledge, currently, there exists no memory anomaly detector designed for OpenMP applications. Since manually detecting memory anomalies is time-consuming, memory anomaly detectors are indispensable when testing an OpenMP application. Our study provides an overview of memory anomalies in OpenMP applications, which can serve as guidance for tool developers in future development.

### Bug Studies

There have been several bug studies on OpenMP. Münchhalfen et al. conducted a study on OpenMP 4.0 and proposed a classification of OpenMP usage errors [17]. The study comprehensively scrutinized the semantics of OpenMP constructs, including tasking and target constructs, illustrating potential incorrect usages and discussing their harmful effects on the program execution. These usage errors are further categorized into *syntactic defects*, *semantic defects*, and *performance defects* according to each error’s root cause and runtime behavior. However, this study only covered a subset of memory anomalies (UUM and USD on the accelerator), and did not provide any concrete examples.

By analyzing data races in real-world OpenMP applications, Liao et al. created DataRaceBench, a micro-benchmark suite designed to evaluate the effectiveness of data race detectors [12]. DataRaceBench contains more than 100 micro-benchmarks with and without data races. Through the provided evaluation script, DataRaceBench can automatically calculate the precision, recall, and accuracy of a data race detector. To further improve the OpenMP standard coverage of DataRaceBench, Liao et al. carried out a study on OpenMP 4.5. They classified OpenMP constructs and clauses into three categories, *parallel semantics*, *shared semantics*, and *synchronization semantics*. Each category is summarized as a group of semantics labels. For any uncovered label, a new micro-benchmark is added to DataRaceBench to enhance coverage.

Apart from OpenMP, there also exist bug studies targeting other programming languages. Lu et al. conducted a concurrency bug study on real-world applications [15]. They examined 105 randomly selected real-world concurrency bugs from four open-source server and client applications. Wang et al. conducted a concurrency bug study on Node.js [25]. They collected 57 real bug cases from GitHub and organized them into a benchmark suite. Nong et al. conducted a comparison of five state-of-art memory anomaly detectors [19]. They used 520 C/C++ programs to measure the effectiveness and efficiency of these detectors. The result indicates that the effectiveness of these detectors varies widely due to the applied detection technique, while the performance is similar. Although all three studies are not related to OpenMP, we picked up some analysis methods when carrying out our study.

## Conclusion

In this paper, we presented a study of memory anomalies that can occur in OpenMP programs. Memory anomalies are common errors in C/C++ applications. For OpenMP applications, memory anomalies may arise if programmers specify erroneous data-sharing attributes or map-types. We have compared the effectiveness of three state-of-the-art memory anomaly detectors. The evaluation results indicate that no single tool can detect all memory anomalies in an OpenMP application.

For future research, we plan to compare the performance of memory anomaly detectors on OpenMP applications. We also plan to extend current tools with new detection algorithms.

## Footnotes

1 Iteration variable is an exception. Its data-sharing attribute can be overwritten to private or last-private, according to the enclosing construct.

2 https://github.com/FuriousBerserker/MemoryAnomalyBench.
